# Administration of URB597, Oleoylethanolamide or Palmitoylethanolamide Increases Waking and Dopamine in Rats

**DOI:** 10.1371/journal.pone.0020766

**Published:** 2011-07-14

**Authors:** Eric Murillo-Rodríguez, Marcela Palomero-Rivero, Diana Millán-Aldaco, Oscar Arias-Carrión, René Drucker-Colín

**Affiliations:** 1 Laboratorio de Neurociencias Moleculares e Integrativas, Escuela de Medicina, División Ciencias de la Salud, Universidad Anáhuac Mayab, Mérida, Yucatán, México; 2 División de Neurociencias, Instituto de Fisiología Celular, Universidad Nacional Autónoma de México, México DF, México; 3 Department of Neurology, Philipps-Universität, Marburg, Germany; Sapienza University of Rome, Italy

## Abstract

**Background:**

Oleoylethanolamide (OEA) and palmitoylethanolamide (PEA) are amides of fatty acids and ethanolamine named *N*-acylethanolamines or acylethanolamides. The hydrolysis of OEA and PEA is catalyzed by the fatty acid amide hydrolase (FAAH). A number of FAAH inhibitors that increase the levels of OEA and PEA in the brain have been developed, including URB597. In the present report, we examined whether URB597, OEA or PEA injected into wake-related brain areas, such as lateral hypothalamus (LH) or dorsal raphe nuclei (DRN) would promote wakefulness (W) in rats.

**Methodology and Principal Findings:**

Male Wistar rats (250–300 g) were implanted for sleep studies with electrodes to record the electroencephalogram and electromyogram as well as a cannulae aimed either into LH or into DRN. Sleep stages were scored to determine W, slow wave sleep (SWS) and rapid eye movement sleep (REMS). Power spectra bands underly neurophysiological mechanisms of the sleep-wake cycle and provide information about quality rather than quantity of sleep, thus fast Fourier transformation analysis was collected after the pharmacological trials for alpha (for W; α = 8–12 Hz), delta (for SWS; δ = 0.5–4.0 Hz) and theta (for REMS; θ = 6.0–12.0 Hz). Finally, microdialysis samples were collected from a cannula placed into the nucleus accumbens (AcbC) and the levels of dopamine (DA) were determined by HPLC means after the injection of URB597, OEA or PEA. We found that microinjection of compounds (10, 20, 30 µg/1 µL; each) into LH or DRN during the lights-on period increased W and decreased SWS as well as REMS and enhanced DA extracellular levels.

**Conclusions:**

URB597, OEA or PEA promoted waking and enhanced DA if injected into LH or DRN. The wake-promoting effects of these compounds could be linked with the enhancement in levels of DA and indirectly mediated by anandamide.

## Introduction

Amides of long-chain fatty acids with ethanolamine are a family of lipids mediators produced through the action of two enzymes: *N*-acyl-transferase and phospholipase D [1], [2]. Fatty acids with ethanolamine (FAE) can be hydrolyzed by the fatty acid amide hydrolase (FAAH; [3]). To this date, a number of FAAH inhibitors have been described [2], [3], including URB597. Several reports have indicated that URB597 inhibits FAAH activity *in vitro* rat brain membranes with an IC_50_ value of 5 nM. Likewise this drug has a remarkable selectivity for FAAH with no activity on other cannabinoid-related elements [4]–[8].

Multiple physiological roles for FAE have been proposed. For instance, oleoylethanolamide (OEA) is related with mechanisms of satiety [9], [10], activates a PPAR-α receptors [11] and it has been related with fat ingestion [12] whereas palmitoylethanolamide (PEA) acts as an antinociceptive molecule [13], [14] and displays anti-inflammatory properties [15].

Previously, we have shown that intracerebroventricular (icv) injections of URB597, OEA or PEA in rats increase alertness, enhance dopamine (DA) and induce *c*-Fos expression in wake-related brain areas, such as lateral hypothalamus (LH) or dorsal raphe nuclei (DRN) [16]. This result prompted us to question whether these compounds might enhance waking if injected directly into these two brain areas linked with the sleep-wake cycle modulation [17]–[19].

A second aim of the present study was to determine if microinjections of URB597, OEA or PEA would increase DA levels collected from nucleus accumbens (AcbC) if administered into LH or DRN. The AcbC was selected as a target for collection of DA due its importance in the modulation of the sleep-wake cycle [20]–[22]. Thus, it was reasonable to hypothesize whether URB597, OEA or PEA would increase waking if injected into LH or DRN and these compounds may enhance the contents of DA collected from AcbC.

## Results

**Figure 1:**
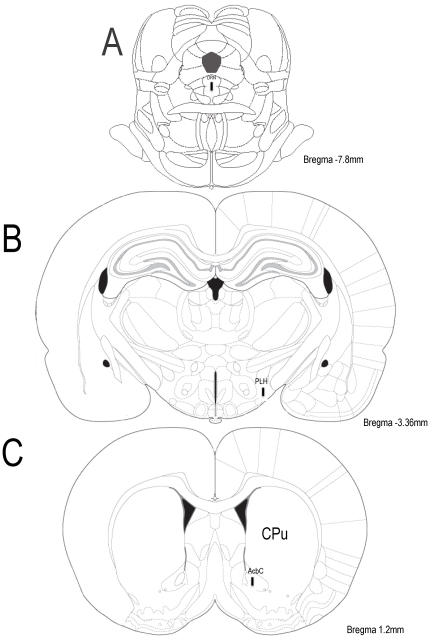
Schematic drawings from rat brain atlas [**23**] showing a vertical black bar that represents the localization of the cannulae placed at DRN (Panel A), LH (Panel B) or the microdialysis probe placed into AcbC (Panel C). Abbreviations: AcbC, nucleus accumbens, core; CPU, caudate putamen; DRN, dorsal raphe nucleus, dorsal part; PLH, peduncular part of lateral hypothalamus.

Figure 1 display schematic drawings from the rat brain atlas [23] showing the localization of the cannulae placed at DRN (Panel A), LH (Panel B) or the microdialysis probe position into AcbC (Panel C). Rats whose cannulae or microdialysis probe placements fell outside of the target areas were excluded from further analysis.

### Effects on sleep after the microinjection of URB597, OEA or PEA into LH

**Figure 2:**
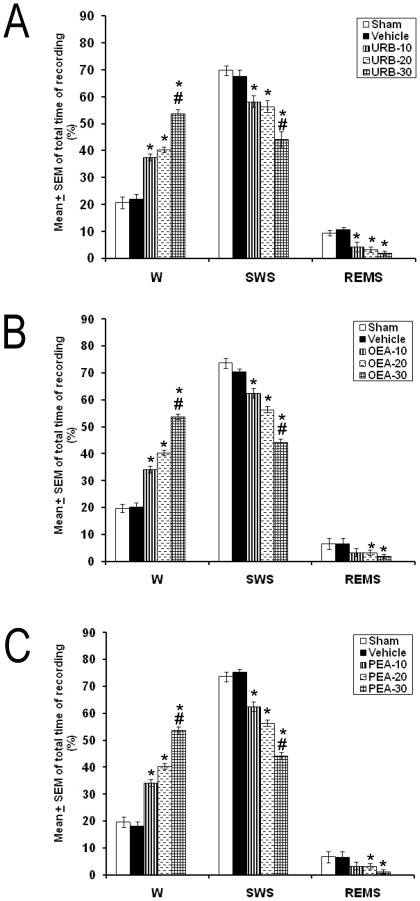
Effects on total time (3 h of sleep recordings) of wakefulness (W), slow wave sleep (SWS) and rapid eye movement sleep (REMS) after administrations into the lateral hypothalamus of either URB597 (Panel A), OEA (Panel B) or PEA (Panel C). Pharmacological treatments (10, 20, 30 µg/1 µL; each compound) increased W and diminished SWS as well as REMS (Mean ± SEM of total time of recording [%]; * vs. Sham/Vehicle, *p*<0.05; # vs. respective compound at 10 or 20 µg/1 µL, *p*<0.05).

In the whole report, no statistical differences were found among sham and VEH groups. Next, in experiment 1, URB597 (10, 20, or 30 µg/1 µL) injected into LH increased W (*p*<0.001) and decreased SWS (*p*<0.001) and REMS (*p*<0.001; Figure 2A). A dose-dependent effect was found in W and SWS after microinjection of URB597. In the OEA intrahypothalamic trial (10, 20, or 30 µg/1 µL; Figure 2B), waking was enhanced (*p*<0.001) whereas SWS (*p*<0.001) and REMS (*p*<0.001) were diminished. Furthermore, a dose-dependent response in W and SWS using OEA was observed. Similarly, PEA injected into LH enhanced waking (*p*<0.01) but decreased SWS (*p*<0.01) and REM (*p*<0.01; Figure 2C).

### Effects on sleep after the microinjection of URB597, OEA or PEA into DRN

**Figure 3:**
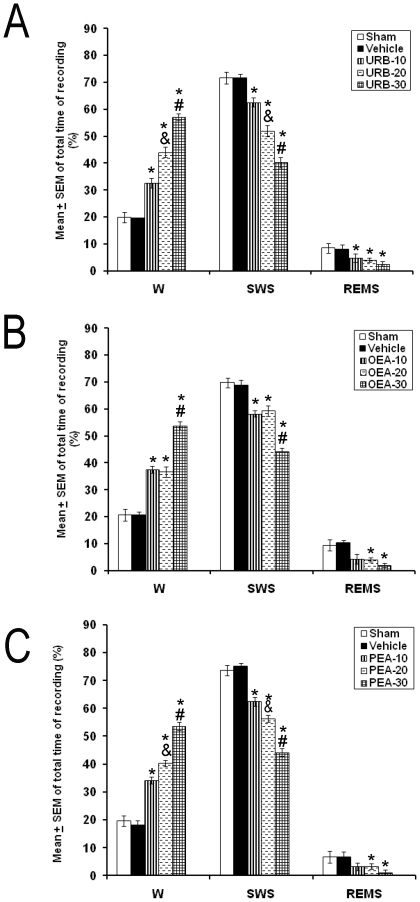
Effects on total time (3 h of sleep recordings) of W, SWS and REMS after administrations into the dorsal raphe nuclei of either URB597 (Panel A), OEA (Panel B) or PEA (Panel C). Pharmacological treatments (10, 20, 30 µg/1 µL; each compound) increased W and diminished SWS as well as REMS (Mean ± SEM of total time of recording [%]; * vs. Sham/Vehicle, *p*<0.05; # vs. respective compound at 10 or 20 µg/1 µL, *p*<0.05; & vs. respective compound at 10 or 20 µg/1 µL, *p*<0.05).

In the next experiment, injection of URB597 (10, 20, or 30 µg/1 µL; Figure 3A) into DRN promoted waking (*p*<0.001) and diminished SWS (*p*<0.001) and REMS (*p*<0.001). Administration of OEA or PEA into DRN (10, 20, or 30 µg/1 µL; each compound) enhanced W (*p*<0.01) and diminished SWS (*p*<0.01) as well as REMS (*p*<0.01; Figure 3B and Figure 3C, respectively). We also found that URB597 and PEA induced a dose-dependent effect in W and SWS.

### Effects on power spectra after the microinjection of URB597, OEA or PEA into LH

**Figure 4:**
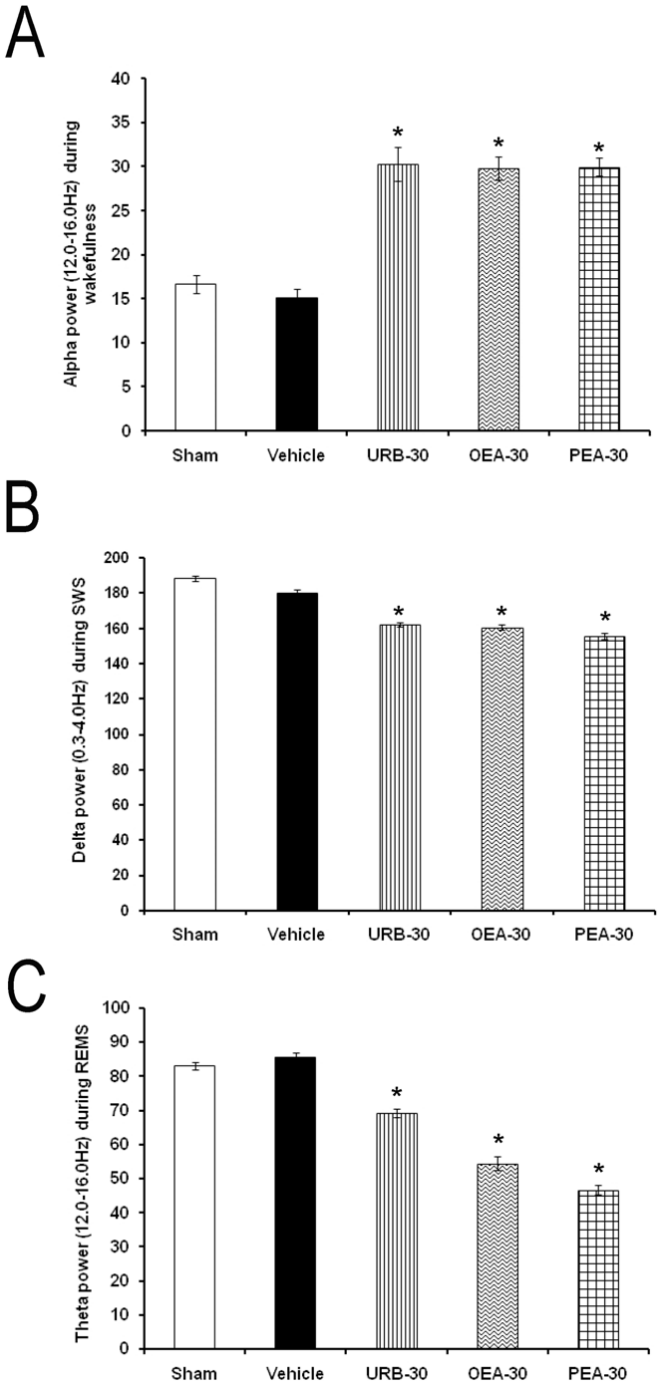
EEG alpha (for W; α = 8–12 Hz. Panel A), delta (for SWS; δ = 0.5–4.0 Hz. Panel B) and theta (for REMS; θ = 6.0–12.0 Hz. Panel C) power spectra (units, µV^2^) in rats after the following treatments into lateral hypothalamus: Sham, vehicle, URB597, OEA or PEA (30 µg/1 µL; each compound). Data were calculated over 3 consecutive hours at every time point (Mean ± SEM; * vs. Sham/Vehicle, *p*<0.05).

Current evidence suggest that power spectra bands provide information about quality rather than quantity of sleep [24], thus we analyzed fast Fourier transformation for alpha (for W; α = 8–12 Hz), delta (for SWS; δ = 0.5–4.0 Hz) and theta (for REMS; θ = 6.0–12.0 Hz) after the pharmacological challenges. Injections into LH of the highest dose of the compounds (30 µg/1 µL; each compound) increased alpha (Figure 4A; *p<*0.05) whereas diminished delta (Figure 4B; *p*<0.05) and theta power (Figure 4C; *p*<0.05).

### Effects on power spectra after the microinjection of URB597, OEA or PEA into DRN

**Figure 5:**
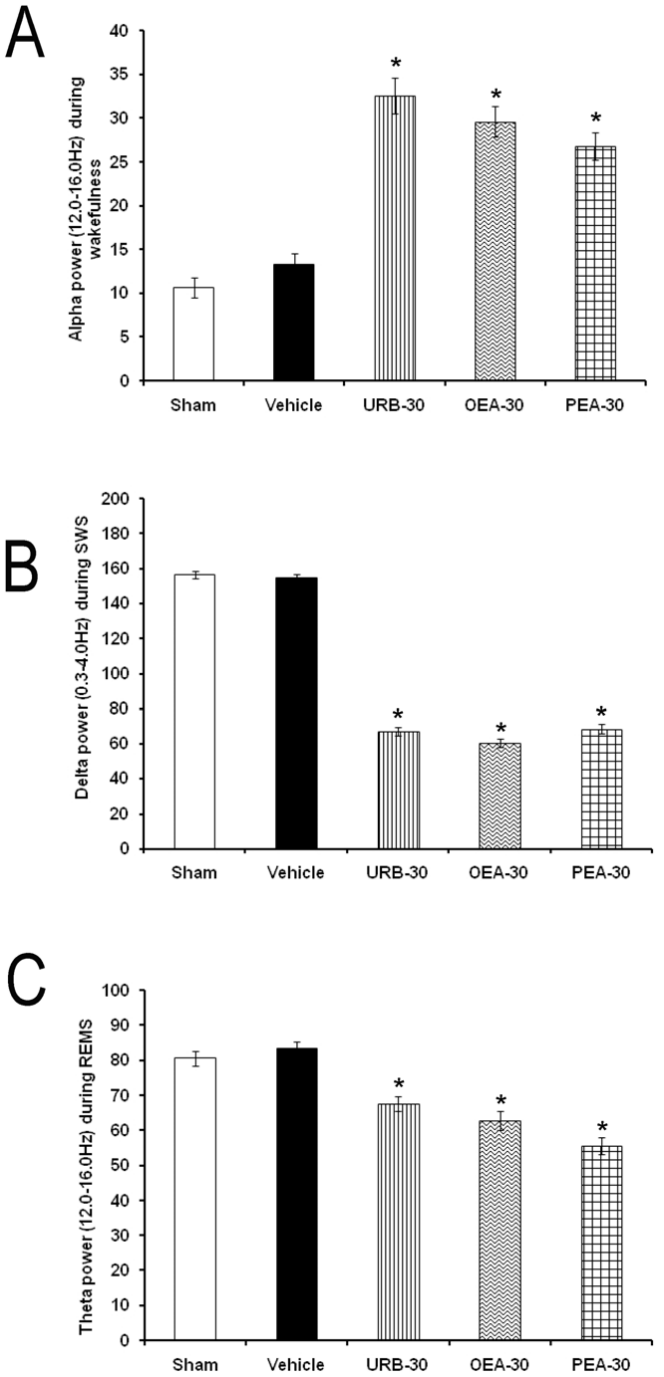
EEG alpha (for W; α = 8–12 Hz. Panel A), delta (for SWS; δ = 0.5–4.0 Hz. Panel B) and theta (for REMS; θ = 6.0–12.0 Hz. Panel C) power spectra (units, µV^2^) in rats after the following treatments into dorsal raphe nuclei: Sham, vehicle, URB597, OEA or PEA (30 µg/1 µL; each compound). Data were calculated over 3 consecutive hours at every time point (Mean ± SEM; * vs. Sham/Vehicle, *p*<0.05).

We next sought to determine whether the injection of the drugs (30 µg/1 µL; each compound) into DRN would induce significant changes in power spectra. It was found that pharmacological trials enhanced alpha (Figure 5A; *p*<0.05) but diminished delta (Figure 5B; *p*<0.05) and theta power (Figure 5C; *p*<0.05).

### Effects on dopamine extracellular levels after the microinjection of URB597, OEA or PEA into LH

**Figure 6:**
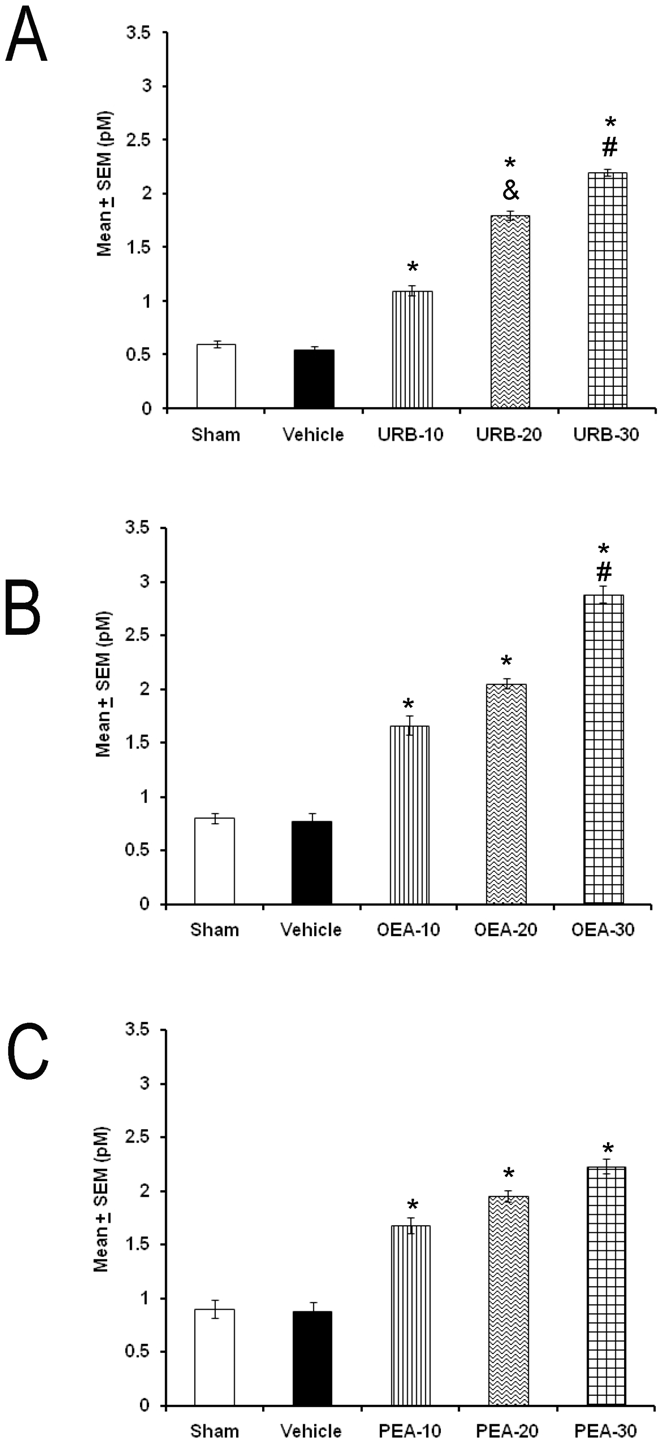
Extracellular levels of DA measured from AcbC during 3 h after the administration into lateral hypothalamus of URB597, OEA or PEA (10, 20, 30 µg/1 µL; each compound). Each point represents Mean ± SEM of pM (* vs. Sham/Vehicle, *p*<0.05; # vs. respective compound at 10/20 µg/1 µL, *p*<0.05; & vs. respective compound at 10 or 20 µg/1 µL, *p*<0.05).

Next, we asked whether microinjections of URB597, OEA or PEA (10, 20, 30 µg/1 µL; each compound) into LH may promote an increase in the DA levels collected from AcbC. Concretely, URB597 enhanced the DA contents (Figure 6A; *p*<0.01) whereas OEA (Figure 6B; *p*<0.05) or PEA (Figure 6C; *p*<0.05) mimicked these effects. Noteworthy, URB597 induced a dose-dependent effect.

### Effects on dopamine extracellular levels after the microinjection of URB597, OEA or PEA into DRN

**Figure 7:**
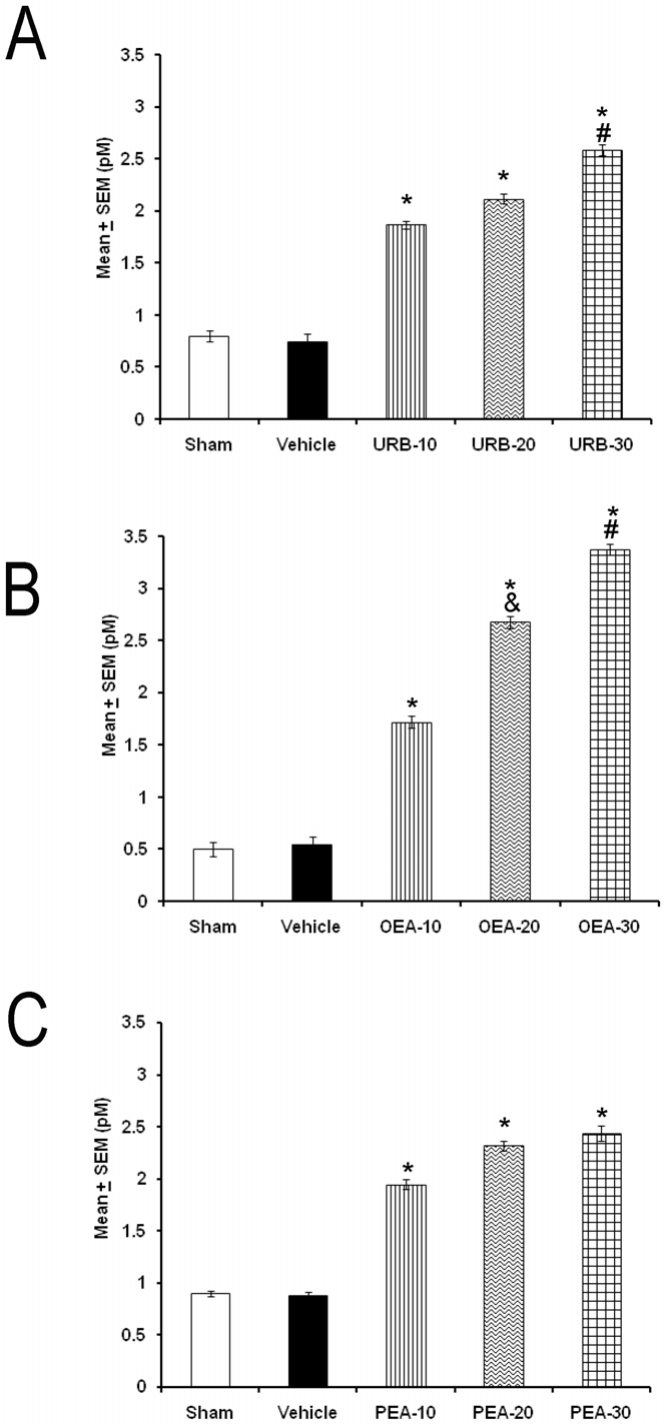
Extracellular levels of DA measured from AcbC during 3 h period after the administration into dorsal raphe nuclei of URB597, OEA or PEA (10, 20, 30 µg/1 µL; each compound). Each point represents Mean ± SEM of pM (* vs. Sham/Vehicle, *p*<0.05; # vs. respective compound at 10/20 µg/1 µL, *p*<0.05; & vs. respective compound at 10 or 20 µg/1 µL, *p*<0.05).

Finally, we tested whether injection of drugs (10, 20, 30 µg/1 µL; each compound) into DRN could induce a significant increase in the extracellular levels of DA collected from AcbC. As predicted, URB597 enhanced the DA contents (Figure 7A; *p*<0.001) and this effect was mimicked by OEA (Figure 7B, *p*<0.001) or PEA (Figure 7C; *p*<0.001). We also found that OEA induced a dose-dependent effect.

## Discussion

The family of *N*-acylethanolamines includes the anoretic mediator OEA, the anti-inflammatory component PEA, and the first endocannabinoid to be described ANA. These compounds are inactivated by the enzymatic hydrolysis, a process that is catalyzed by the FAAH [1]–[3], [9]–[15]. In recent years, different FAAH inhibitors have been developed, such as URB597 [4]–[8].

The current study describes that microinjections of URB597, OEA or PEA into wake-promoting brain areas, such as LH or DRN, promote alertness and enhance DA levels. A lingering question is whether the effects observed in the current report would be mediated by ANA since the inactivation of FAAH by URB597 increases the levels of this endocannabinoid. The wake-promoting effects caused by URB597 might not likely caused by elevated ANA levels since the endocannabinoid system has been linked with sleep promotion. It is worth noting that the activation of the CB_1_ cannabinoid receptor by the antagonist, SR141716A, decreases sleep [25], systemic or central injections of ANA promote sleep [26]–[28], higher levels of ANA have been described in sleep-related brain regions [29], and the ANA membrane transporter blocker, VDM-11, administered in rats promotes sleep [30].

If URB597 increases levels of OEA, PEA and ANA, and this last compound enhances sleep, how we can explain that injection of the FAAH inhibitor induced waking? This discrepancy could be attributed to the following possibility: Indeed, administrations of URB597 enhance endogenous levels of ANA but with higher rates for OEA and PEA [6]. It is therefore possible to conjecture that ANA and URB597 could be promoting opposite effects in sleep by activating unknown brain mechanisms. It should be noted that icv injections of the two ANA congeners, OEA or PEA, promote waking [16]. Furthermore, endogenous levels of OEA and PEA have been described higher in wake-related brain nuclei such as pons and hypothalamus during the active period of the rat [29]. Aforementioned contributions to the role of OEA or PEA on sleep-wake cycle seem to be favorable in terms of wake-modulating properties.

An important question that remains to be addressed pertains to the diffusion of URB597, OEA or PEA after injection. Solely based on the obtained data and representing as a limitation of the study, we are not able to exclude that the compounds are not diffusing to other brain regions. Because our results indicate that URB597, OEA or PEA enhanced alertness as well as DA levels, it could be speculated that if the drugs are diffusing and activating other brain areas, it may be either LH or DRN vicinity. However, it is tempting to hypothesize that the effects observed in this study could be mediated by the activity of neurons placed in LH or DRN. In this regard, it is known that both brain areas are key elements in the modulation of waking [17]–[23], [31].

The effects of the trials on alpha, delta and theta power spectra are also significant. Since the EEG power gauges the potency of multiple cortical-subcortical neuronal networks along different firing frequencies [24], it is possible that the increase in the EEG alpha power may reflect higher neuronal synchrony activated by URB597, OEA or PEA. Nevertheless, the decrease in delta and theta power in SWS and REM sleep, respectively, would suggest a deficiency in sleep consolidation. Further experiments are needed to determine whether effects in EEG power spectra can be related with changes in activity of neurons related with generation of power spectra.

The current study demonstrates that injection of URB597, OEA or PEA either into LH or DRN increases DA levels collected from AcbC. It is conceivable that these compounds could be increasing alertness by enhancing DA levels. Several studies have come to the conclusions that the axis accumbens-hypothalamus-DRN plays a role in sleep modulation. For example, previous studies have shown neuroanatomical projections from LH and DRN to AcbC [32]–[34], the role of these brain nuclei on sleep modulation [17]–[19], [22], [31] and yet the importance of DA in waking has long been recognized [35].

In summary, injection of the FAAH inhibitor, URB597, as well as the endogenous lipids, OEA and PEA promotes waking if injected into wake-promoting brain area such as LH or DRN. Furthermore, these compounds enhance the extracellular levels of DA collected from AcbC. Despite that the current study has several limitations and needs confirmation by performing larger studies; it provides a framework for understanding the neurobiological functions of FAAH as well as endogenous lipids such as OEA and PEA on sleep modulation.

## Materials and Methods

Male Wistar rats (250–300 g) were housed at constant temperature (21±1°C) and under a controlled light-dark cycle (lights on: 07:00–19:00 h). All procedures were conducted in accordance with the Mexican Institutes of Health Research (DOF. NOM-062-Z00-1999) as well as the National Institutes of Health Guide for the Care and Use of Laboratory Animals (NIH publication No. 80-23, revised 1996) and the experimental protocol was approved by the Committee on the Ethics of Animal Experiments of our Institutions. All efforts were made to minimize animal suffering, and to reduce the number of rats used. Compounds were kindly provided by Professor Daniele Piomelli (University of California, Irvine. USA) and were dissolved in vehicle (VEH; composed of polyethylglycol/saline; 5∶95 v/v).

Animals (n = 8) were implanted for sleep studies with electrodes to record the electroencephalogram (EEG) and electromyogram (EMG) as well as a cannulae (23gauge) aimed either into LH (A = −3.3; L = ±1.6; H = −8.2 mm [23]) or into DRN, dorsal part (A = −7.8; L = +0.2; H = −7.1 [23]). The EEG/EMG data was scored in 12 s epochs to determine W, SWS and REMS with the aid of a sleep-scoring program (ICELUS). Power spectra bands underly neurophysiological mechanisms of the sleep-wake cycle and provide information about quality rather than quantity of sleep, therefore, fast Fourier transformation analysis was collected for alpha (for W; α = 8–12 Hz), delta (for SWS; δ = 0.5–4.0 Hz) and theta (for REMS; θ = 6.0–12.0 Hz). The sleep and power spectra data were obtained during that period of time and were analyzed as previously reported [30], [36].

For the microdialysis experiment, a different group of rats (n = 8) was implanted with a guide-cannula (IC guide. BioAnalytical Systems, West Lafayette, IN, USA) into AcbC (target coordinates: A = +1.2; L = 2.0; H = −7.0 [23]) as well as a cannulae (23gauge) aimed either into LH or into DRN (coordinates described above). The microdialysis collection sample procedure and neurochemical analysis for DA was developed as previously reported [16]. Due that we have reported that URB597, OEA and PEA modify sleep within a time frame of 3 h [16], sleep data and microdialysis samples were collected exclusively during the same period of time.

At the beginning of the lights-on period (07:00 h), pharmacological trials were administered randomly as follows: VEH (n = 8), URB597 (n = 8), OEA (n = 8) or PEA (n = 8). Different doses of each compound were used (10, 20, 30 µg/1 µL) and to determine whether the injection of VEH could modify the sleep-wake cycle or the DA contents, an additional group (sham; n = 8) was included. In the whole study, injections were carried out slowly over 1 µL/min.

Results are expressed as mean ± SEM and the significance of differences between groups was evaluated by one-way analysis of variance (ANOVA) followed by the Scheffé's *post-hoc* test (STATVIEW). Differences were considered significant if *p*<0.05.

## References

[1] Schmid HH, Schmid PC, Natarajan V (1996). The N-acylation-phosphodiesterase pathway and cell signalling.. Chem Phys Lipids.

[2] Hansen HS (2010). Palmitoylethanolamide and other anandamide congeners. Proposed role in the diseased brain.. Exp Neurol.

[3] Ahn K, McKinney MK, Cravatt BF (2008). Enzymatic pathways that regulate endocannabinoid signaling in the nervous system.. Chem Rev.

[4] Kathuria S, Gaetani S, Fegley D, Valiño F, Duranti A (2003). Modulation of anxiety through blockade of anandamide hydrolysis.. Nat Med.

[5] Mor M, Rivara S, Lodola A, Plazzi PV, Tarzia G (2003). Cyclohexylcarbamic acid 3′- or 4′-substituted biphenyl-3-yl esters as fatty acid amide hydrolase inhibitors: synthesis, quantitative structure-activity relationships, and molecular modeling studies.. J Med Chem.

[6] Fegley D, Gaetani S, Duranti A, Tontini A, Mor M (2005). Characterization of the fatty acid amide hydrolase inhibitor cyclohexyl carbamic acid 3′-carbamoyl-biphenyl-3-yl ester (URB597): effects on anandamide and oleoylethanolamide deactivation.. J Pharmacol Exp Ther.

[7] Piomelli D, Tarzia G, Duranti A, Tontini A, Mor M (2006). Pharmacological profile of the selective FAAH inhibitor KDS-4103 (URB597).. CNS Drug Rev.

[8] Tarzia G, Duranti A, Tontin A, Piersanti G, Mor M (2003). Design, synthesis, and structure-activity relationships of alkylcarbamic acid aryl esters, a new class of fatty acid amide hydrolase inhibitors.. J Med Chem.

[9] Rodríguez de Fonseca F, Navarro M, Gómez R, Escuredo L, Nava F (2001). An anorexic lipid mediator regulated by feeding.. Nature.

[10] Gaetani S, Oveisi F, Piomelli D (2003). Modulation of meal pattern in the rat by the anorexic lipid mediator oleoylethanolamide.. Neuropsychopharmacol.

[11] Fu J, Gaetani S, Oveisi F, Lo Verme J, Serrano A (2003). Oleylethanolamide regulates feeding and body weight through activation of the nuclear receptor PPAR-alpha.. Nature.

[12] Schwartz GJ, Fu J, Astarita G, Li X, Gaetani S (2008). The lipid messenger OEA links dietary fat intake to satiety.. Cell Metab.

[13] Calignano G, La Rana G, Piomelli D (2001). Antinociceptive activity of the endogenous fatty acid amide, palmitylethanolamide.. Eur J Pharmacol.

[14] Capasso R, Izzo AA, Fezza F, Pinto A, Capasso F (2001). Inhibitory effect of palmitoylethanolamide on gastrointestinal motility in mice.. Br J Pharmacol.

[15] Lo Verme J, Fu J, Astarita G, La Rana G, Russo R (2005). The nuclear receptor peroxisome proliferator-activated receptor-alpha mediates the anti-inflammatory actions of palmitoylethanolamide.. Mol Pharmacol.

[16] Murillo-Rodríguez E, Vázquez E, Millán-Aldaco D, Palomero-Rivero M, Drucker-Colín D (2007). Effects of the fatty acid amide hydrolase inhibitor URB597 on the sleep-wake cycle, c-Fos expression and dopamine levels of the rat.. Eur J Pharmacol.

[17] Jones BE (2005). From waking to sleeping: neuronal and chemical substrates.. Trends Pharmacol Sci.

[18] Llinás RR, Steriade M (2006). Bursting of thalamic neurons and states of vigilance.. J Neurophysiol.

[19] Steriade M (2006). Grouping of brain rhythms in corticothalamic systems.. Neurosci.

[20] Dzirasa K, Ribeiro S, Costa R, Santos LM, Lin SC (2006). Dopaminergic control of sleep-wake states.. J Neurosci.

[21] Monti JM, Monti D (2007). The involvement of dopamine in the modulation of sleep and waking.. Sleep Med Rev.

[22] Jones BE (2005). From waking to sleeping: neuronal and chemical substrates.. Trends Pharmacol Sci.

[23] Paxinos G, Watson C (2005). The Rat Brain in Stereotaxic Coordinates.

[24] Corsi-Cabrera M, Pérez-Garci E, Del Rio-Portilla Y, Ugalde E, Guevara MA (2001). EEG bands during wakefulness, slow-wave, and paradoxical sleep as a result of principal component analysis in the rat.. Sleep.

[25] Santucci V, Storme JJ, Soubrié P, Le Fur G (1996). Arousal-enhancing properties of the CB1 cannabinoid receptor antagonist SR 141716A in rats as assessed by electroencephalographic spectral and sleep-waking cycle analysis.. Life Sci.

[26] Murillo-Rodríguez E, Sánchez-Alavez M, Navarro L, Martínez-González D, Drucker-Colín R (1998). Anandamide modulates sleep and memory in rats.. Brain Res.

[27] Murillo-Rodríguez E, Cabeza R, Méndez-Díaz M, Navarro L, Prospéro-García O (2001). Anandamide-induced sleep is blocked by SR141716A, a CB1 receptor antagonist and by U73122, a phospholipase C inhibitor.. Neuroreport.

[28] Murillo-Rodríguez E, Blanco-Centurión C, Sánchez C, Piomelli D, Shiromani PJ (2003). Anandamide enhances extracellular levels of adenosine and induces sleep: an in vivo microdialysis study.. Sleep.

[29] Murillo-Rodríguez E, Désarnaud F, Prospéro-García O (2006). Diurnal variation of arachidonoylethanolamine, palmitoylethanolamide and oleoylethanolamide in the brain of the rat.. Life Sci.

[30] Murillo-Rodríguez E, Millán-Aldaco D, Di Marzo V, Drucker-Colín R (2008). The anandamide membrane transporter inhibitor, VDM-11, modulates sleep and c-Fos expression in the rat brain.. Neurosci.

[31] Murillo-Rodríguez E, Arias-Carrión O, Sanguino-Rodríguez K, González-Arias M, Haro R (2009). Mechanisms of sleep-wake cycle modulation.. CNS Neurol Disord Drug Targets.

[32] Haber SN, Groenewegen HJ, Grove EA, Nauta WJ (1985). Efferent connections of the ventral pallidum: evidence of a dual striato pallidofugal pathway.. J Comp Neurol.

[33] Stratford TR, Wirtshafter D (1990). Ascending dopaminergic projections from the dorsal raphe nucleus in the rat.. Brain Res.

[34] Zahm DS, Heimer L (1993). Specificity in the efferent projections of the nucleus accumbens in the rat: comparison of the rostral pole projection patterns with those of the core and shell.. J Comp Neurol.

[35] Monti JM, Jantos H (2008). The roles of dopamine and serotonin, and of their receptors, in regulating sleep and waking.. Prog Brain Res.

[36] Murillo-Rodríguez E, Millán-Aldaco D, Palomero-Rivero D, Mechoulam R, Drucker-Colín D (2008). The non-psychoactive cannabis constituent cannabidiol is a wake-inducing agent. Behav.. Neurosci.

